# Enhanced water saturation estimation in hydrocarbon reservoirs using machine learning

**DOI:** 10.1038/s41598-025-13982-5

**Published:** 2025-08-14

**Authors:** Ali Akbari, Ali Ranjbar, Yousef Kazemzadeh, Dmitriy A. Martyushev

**Affiliations:** 1 Department of Petroleum Engineering, Faculty of Petroleum, Gas, and Petrochemical Engineering, Persian Gulf University, Bushehr, Iran; 2 Department of Oil and Gas Technologies, Perm National Research Polytechnic University, Perm, Russia 614990

**Keywords:** Water saturation (Sw), Petrophysical analysis, Machine learning (ML), Outlier detection, Hydrocarbon reservoir, Oil recovery optimization, Chemistry, Engineering

## Abstract

Accurate estimation of water saturation (Sw) is essential for optimizing oil recovery strategies and is a key component in petrophysical analyses of hydrocarbon reservoirs. Traditional Sw estimation approaches often face limitations due to idealized assumptions, dependency on core-derived parameters, and geological heterogeneity. In this study, a comprehensive dataset consisting of 30,660 independent data points was utilized to develop machine learning (ML) models for Sw prediction. Nine well log parameters—Depth (DEPT), High-Temperature Neutron Porosity, True Resistivity, Computed Gamma Ray, Spectral Gamma Ray, Hole Caliper, Compressional Sonic Travel Time, Bulk Density, and Temperature—were used as input features to train and test five ML algorithms: Linear Regression, Support Vector Machine (SVM), Random Forest, Least Squares Boosting, and Bayesian methods. To improve model performance, a Gaussian outlier removal technique was applied to eliminate anomalous data points. The models were rigorously validated using multiple training/testing data splits and ten independent runs to ensure statistical reliability. Among the tested models, SVM achieved the highest accuracy, with R^2^ values of 0.9952 (test) and 0.9962 (train) and RMSE values of 0.002 (test) and 0.001 (train). These results demonstrate that ML—particularly SVM—offers a robust and accurate alternative for Sw estimation, supporting more effective reservoir evaluation and oil recovery optimization.

## Introduction

Accurate estimation of water saturation (Sw) is essential for reservoir characterization and plays a significant role in optimizing oil recovery processes^1–8^; as it directly influences reserve estimation, fluid flow modeling, and the efficiency of secondary and enhanced oil recovery (EOR) techniques^9–16^. Traditionally, Sw has been estimated using well log data and empirical models like Archie’s equation and its variations^17–19^; however, these methods are constrained by idealized assumptions, reliance on core-derived parameters, and difficulties in handling complex lithologies and reservoir heterogeneity. Moreover, uncertainties in well log measurements and reservoir conditions further reduce the reliability of such conventional approaches, prompting the need for more advanced, data-driven techniques^20,21^. Well logging data provides critical information on key reservoir properties such as porosity, permeability, and water saturation^22–26^; which are instrumental in analyzing reservoir behavior and enhancing production strategies^27^, particularly in the context of secondary and EOR applications^28–32^. Despite their widespread use, traditional methods grounded in empirical and geological assumptions suffer from significant limitations, making them less dependable under diverse reservoir conditions^33^.

In recent years, machine learning (ML) has emerged as a transformative tool in petrophysical studies, enabling the modeling of complex and nonlinear relationships among reservoir properties that traditional methods often fail to capture^34–38^. By leveraging large datasets, ML techniques improve prediction accuracy, especially under conditions of uncertainty where conventional approaches are less effective^39–41^. Significant advancements have shown the efficacy of ML in subsurface applications such as permeability and porosity prediction, lithofacies classification, and well log interpretation^42–44^, yet limited attention has been given to its application in Sw estimation, particularly with respect to data quality enhancement, outlier removal, and model interpretability^45–47^. ML models offer considerable advantages in Sw prediction due to their ability to process vast datasets, reduce computational costs, and improve accuracy by identifying complex patterns in data^48,49^, often outperforming conventional simulation techniques^50,51^. However, challenges remain, including model complexity, the demand for extensive training data, and limited adaptability across diverse reservoir conditions, highlighting the need for continued development to improve both performance and interpretability. Nonetheless, ML holds strong potential to significantly enhance petrophysical estimation and oil recovery effectiveness in complex and heterogeneous reservoirs.

In 2019, Al-Mudhafar^52^ investigated the importance of integrating discrete lithofacies distributions into petrophysical property modeling to preserve reservoir heterogeneity and enhance flow simulation accuracy. In the South Rumaila oil field, a combination of probabilistic neural networks and smooth generalized additive models (SGAM) was employed to estimate core permeability based on well log data such as neutron porosity, shale volume, and water saturation. By incorporating predicted lithofacies as an independent variable, the SGAM demonstrated superior accuracy over generalized linear models (GLM), particularly by resolving multicollinearity between inputs. In a subsequent study, in 2019, Al-Mudhafar^53^, applied Bayesian Model Averaging (BMA) and LASSO regression to improve core permeability prediction in sandstone formations, achieving accurate results validated by adjusted R^2^ and RMSE metrics. These integrated statistical approaches highlight the potential of ML in addressing reservoir complexity—an approach similarly used in our study for enhancing Sw estimation using SVM, Random Forest, and other ML models, where lithological variations and multivariate dependencies must also be effectively captured.

In 2022, Al-Mudhafar and Wood^54^ extended this application by developing a predictive lithofacies classification model using tree-based ensemble classifiers—XGBoost and AdaBoost—applied to the Mishrif carbonate reservoir. These classifiers, when combined with XGBoost regression, significantly improved the prediction of permeability using well-log inputs including porosity, gamma ray, water saturation, and resistivity. The study achieved high prediction accuracies with total correct percentages (TCP) reaching up to 100% for training and 97% for testing datasets. Similarly, in 2023, Li et al.^55^ employed LightGBM and Random Forest models to predict pore pressure in abnormally pressured reservoir blocks, achieving an R^2^ of 0.935 and over 90% prediction accuracy by using sonic and density logs. These findings confirm the robustness of ensemble learning models in subsurface property prediction tasks. Inspired by these studies, our approach incorporates SVM and ensemble ML algorithms, validated across various training/testing splits, to accurately predict Sw—another critical petrophysical parameter—demonstrating that combining data preprocessing with powerful ML models can yield highly reliable estimations in heterogeneous reservoirs.

Several recent studies further validate the effectiveness of ML in geomechanical and petrophysical property estimation. In 2023, Delavar and Ramezanzadeh^56^ applied hybrid ML techniques, including LSSVM-PSO and RF-Bayesian, for pore pressure prediction in complex carbonate reservoirs, achieving average R^2^ values of 0.97 and 0.96, respectively. In a similar vein, in 2024, Das and Maiti^57^, used decision tree regression combined with empirical approaches to predict overpressure zones in oceanic slope settings, highlighting ML’s capability in complex geological environments. In 2022, Radwan et al.^58^ used six well-log variables and applied various ML algorithms (DT, AdaBoost, RF, TOB) for pore pressure prediction in the Mangahewa gas field, outperforming multiple linear regression, particularly in data-limited scenarios. Likewise, in 2023, Allawi et al.^59^ presented a semi-analytical model to predict rock compressibility in sandstone formations, validated against lab measurements and outperforming existing empirical correlations. These diverse but related efforts demonstrate how ML is transforming subsurface property estimation, supporting our motivation to leverage ML—especially SVM—as a high-accuracy, data-driven approach for improving Sw estimation and ultimately supporting more effective reservoir evaluation and oil recovery planning.

This study introduces a comprehensive and novel workflow for estimating Sw in hydrocarbon reservoirs using advanced ML techniques, particularly Support Vector Machine (SVM), in conjunction with rigorous data preprocessing. Traditional empirical correlations—such as Archie’s equation and its variants—often assume homogenous lithology and fixed rock-fluid interaction parameters, which are rarely valid in heterogeneous reservoir settings. These limitations reduce their accuracy and generalizability across complex geological conditions. To overcome these constraints, the present workflow begins with a robust dataset of well-log parameters (DEPT, HTNP, RT, CGR, SGR, HCAL, DTCO, RHOZ, and TEMP), followed by a Gaussian-based outlier removal method to improve data quality. Five ML algorithms—Linear Regression (LR), SVM, Random Forest (RF), Least Squares Boosting (LSBoost), and Bayesian modeling—were developed and compared in terms of performance. The strength of this workflow lies in its ability to handle nonlinearity, noise, and high-dimensional data while ensuring better generalization to unseen data. Among the tested models, SVM demonstrated superior performance with the highest R2 and lowest RMSE, validating its robustness in modeling complex petrophysical relationships. However, the approach still requires careful tuning of hyperparameters and adequate data volume to achieve reliable predictions across different reservoirs. Overall, this ML-based workflow offers a data-driven and scalable alternative to traditional methods, improving the reliability of Sw estimation and supporting more informed decisions in reservoir characterization and oil recovery planning.

## Methodology

### Data collection & processing

In this study, the data required for developing high-accuracy and efficient ML models were extracted from hydrocarbon fields located in the southwestern region of Iran. The data acquisition process focused on obtaining high-quality, real-field measurements to ensure the reliability and representativeness of the dataset. The dataset includes a broad spectrum of well-log parameters such as SW, DEPT, HTNP, RT, CGR, SGR, HCAL, DTCO, RHOZ, and TEMP, comprising a total of 30,660 individual data points. This comprehensive dataset enables the analysis of variable behavior under different reservoir conditions and allows ML models to effectively predict and evaluate complex petrophysical relationships. Table 1 provides a detailed description of the input features used in this study.

**Table 1:** Input data to ML models*.*

Sw (water saturation)	The fraction of pore volume occupied by water, essential for reservoir evaluation and hydrocarbon estimation
HTNP (high-temperature neutron porosity)	A neutron porosity log adapted for high-temperature conditions, used to estimate porosity in formations
RT (true resistivity)	The formation’s true electrical resistivity, which helps determine hydrocarbon and water saturation
CGR (computed gamma ray)	A gamma-ray log that quantifies natural radioactivity in formations, distinguishing between shale and clean formations
SGR (spectral gamma ray)	A gamma-ray log that differentiates radioactive elements (K, U, Th) to improve lithology interpretation
HCAL (hole caliper)	Measures borehole diameter to assess wellbore stability, washouts, and mudcake thickness
DTCO (compressional sonic travel time)	Measures the interval transit time of compressional waves in rock, used to estimate porosity and mechanical properties
RHOZ (bulk density log)	A density log that provides formation bulk density, crucial for porosity estimation and lithology identification

The dataset used in this study was compiled from various published sources and internal databases to ensure diversity and generalizability of the machine learning models. Although the primary focus was on petrophysical log data, the geological setting of the wells reflects typical characteristics of sandstone-dominated hydrocarbon reservoirs. The reservoir formations include siliciclastic sequences with varying degrees of sorting and compaction, resulting in a wide porosity range from approximately 10 to 25%. These variations are influenced by depth, depositional environment, and cementation effects. The lithology primarily consists of medium to fine-grained sandstones interbedded with shales, which introduces heterogeneity in fluid saturation and log responses. Such geological complexity makes conventional water saturation estimation less reliable and further justifies the application of machine learning techniques to improve prediction accuracy in these heterogeneous formations.

Prior to model training, input features were standardized using z-score normalization to ensure consistent scaling across all variables. This process involved subtracting the mean and dividing by the standard deviation of each feature, resulting in standardized values with zero mean and unit variance. Standardization was especially important for models such as SVM and LR, which are sensitive to feature scale. Furthermore, Pearson correlation analysis was performed to identify potential multicollinearity among input features. Despite moderate correlations between some parameters, all nine features were retained due to their individual importance and non-redundant contributions to the target variable (Sw) prediction.

For data analysis, various tools such as scatter plots, histograms, heatmaps, box plots, and violin plots were utilized. Scatter plots are used to depict the relationship between two numerical variables and can assist in identifying correlations, clusters, and outliers. In contrast, histograms represent the distribution of a numerical variable by dividing the data into intervals and displaying the frequency of each interval as a bar chart. Heatmaps use colors to indicate the intensity or magnitude of data in a two-dimensional matrix, making them highly useful for analyzing correlation matrices and multidimensional data. Box plots summarize data distributions by presenting the median, quartiles, and interquartile range, which are helpful in identifying outliers and comparing distributions. Additionally, violin plots, which combine box plots and density curves, not only show the median and quartiles but also represent the shape of the data distribution, making them particularly useful for detailed analysis and identifying multimodal distributions.

**Fig. 1:**
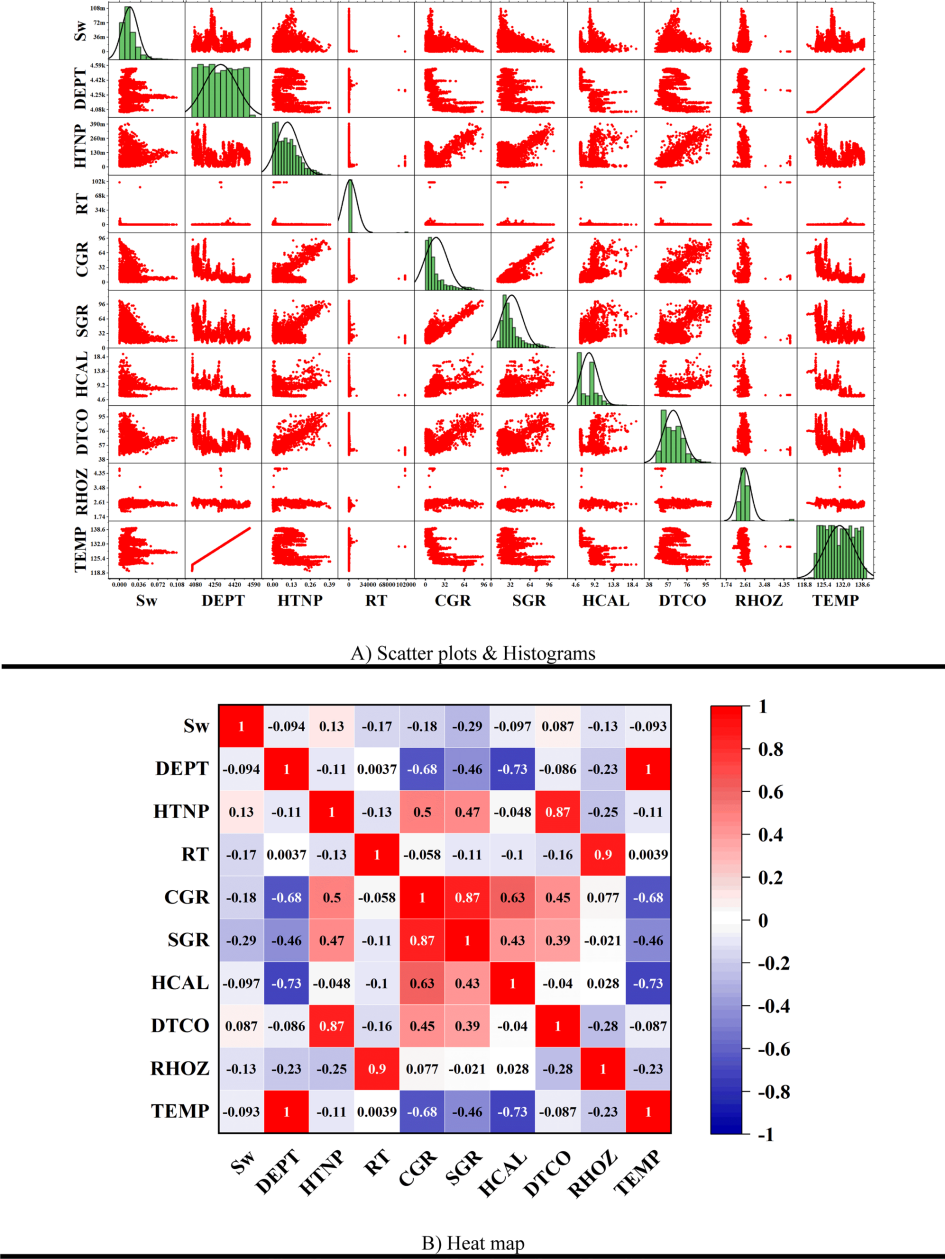

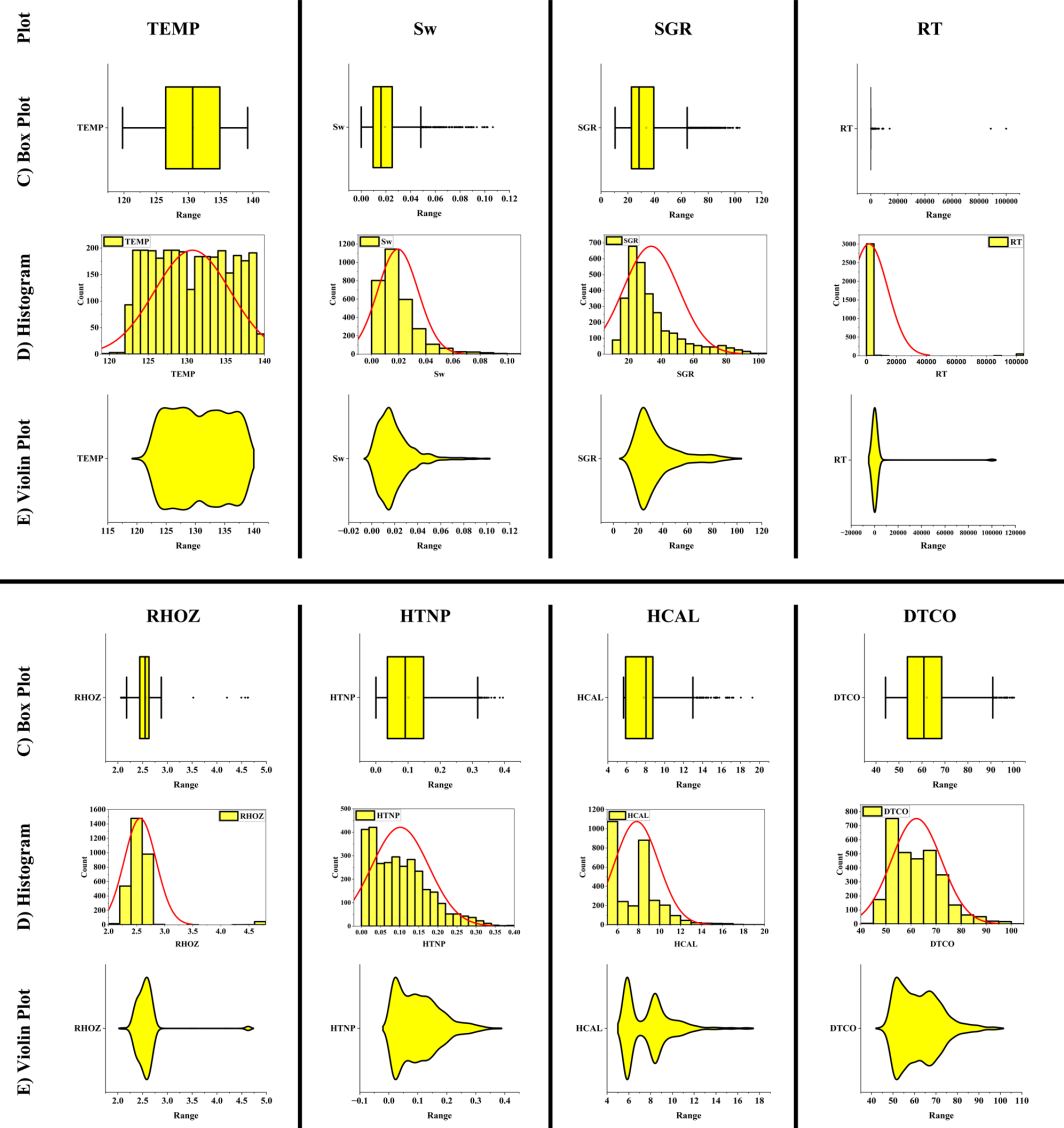

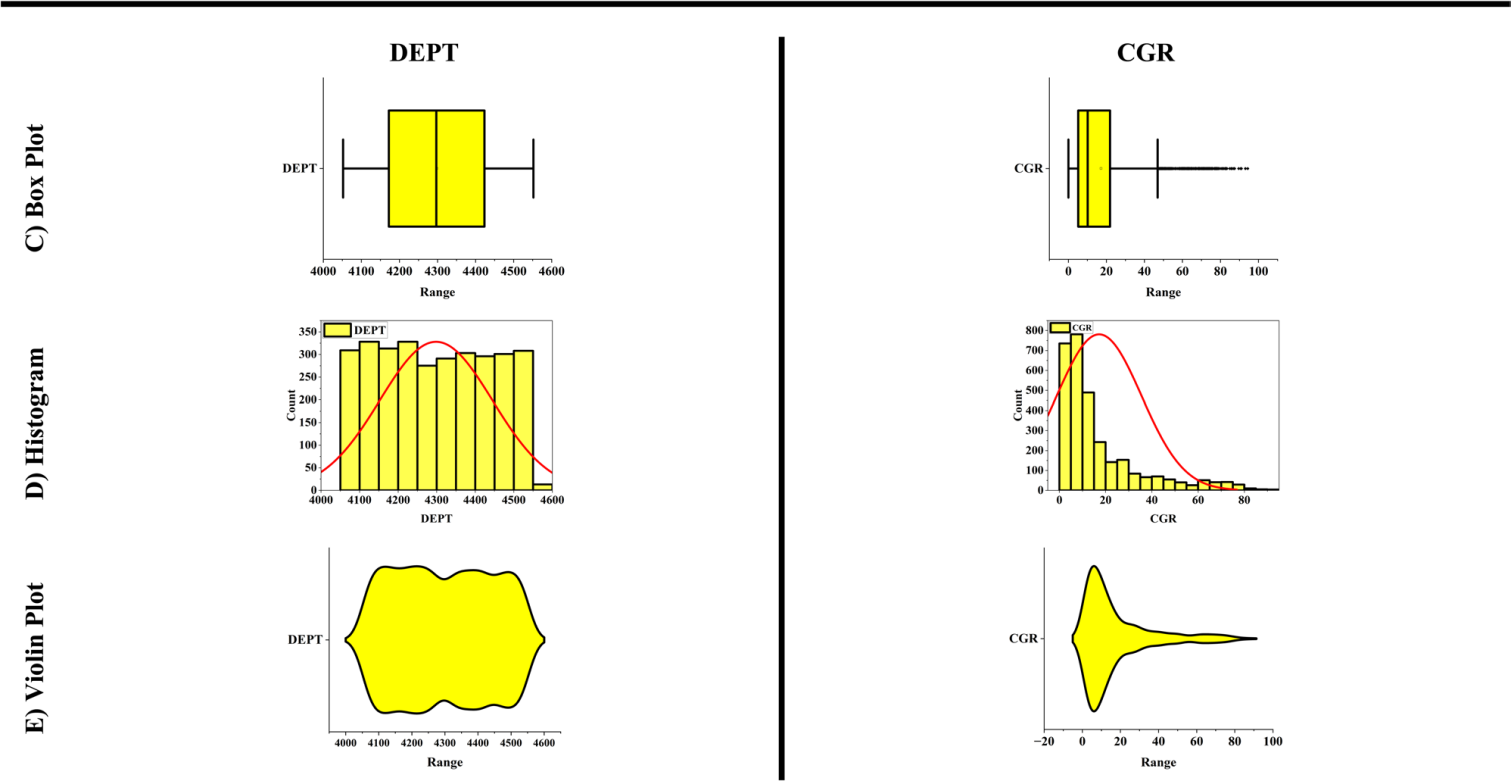
Specialized analysis of input data (before outlier removal). (**A**) Scatter plots & Histograms, (**B**) Heat map, (**C**) Box plots, (**D**) Histograms, (**E**) Violin plots.

**Fig. 2:**
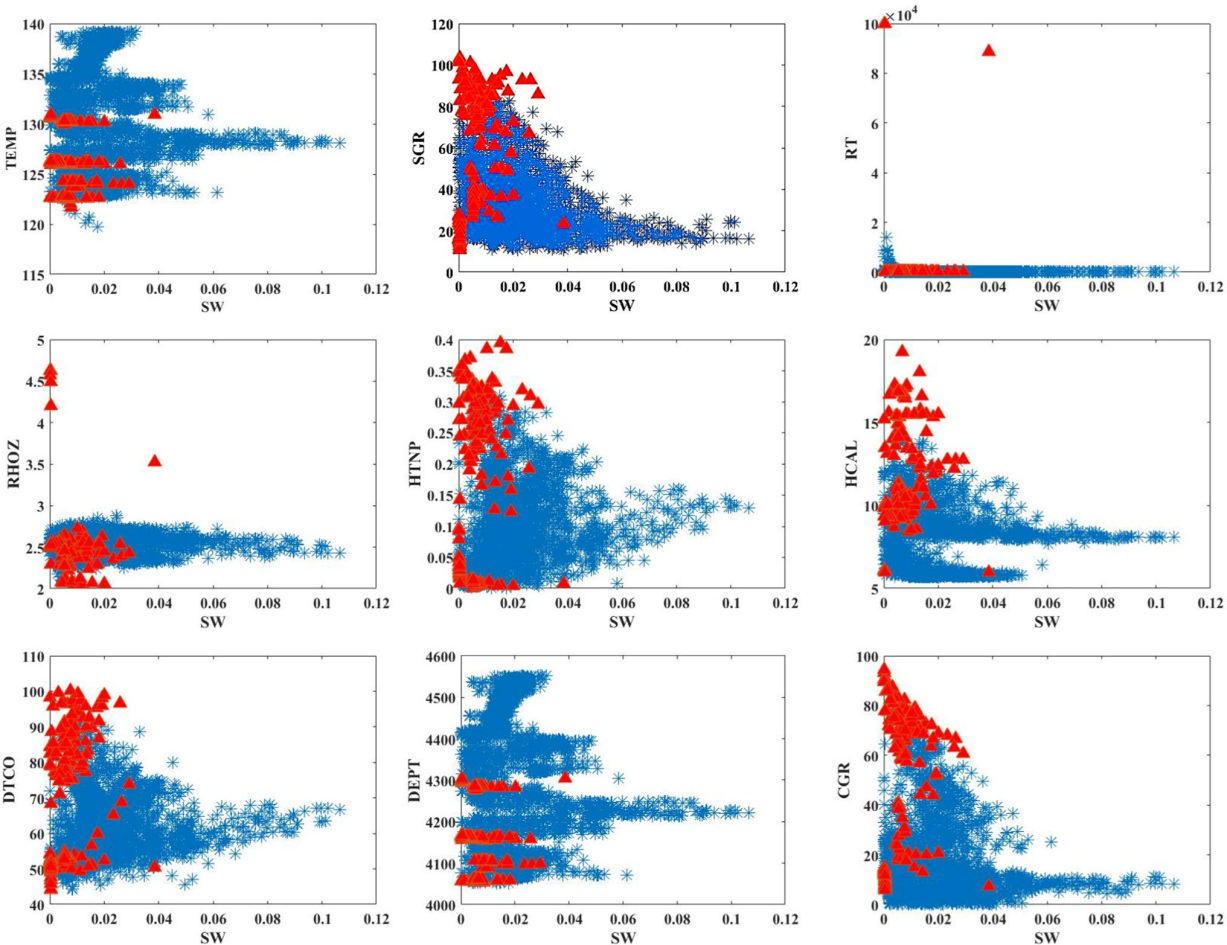
Outlier removal using the Gaussian method.

**Fig. 3:**
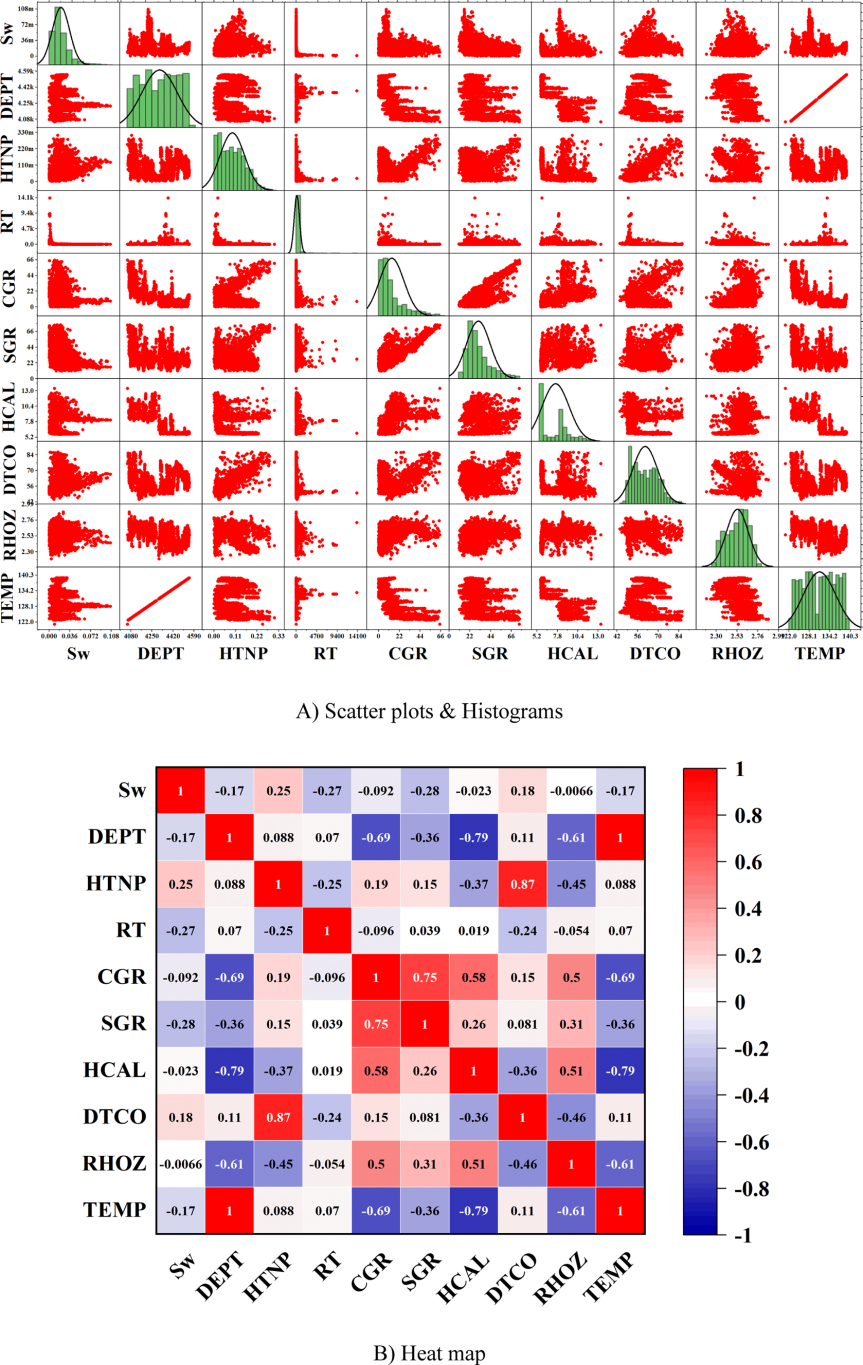

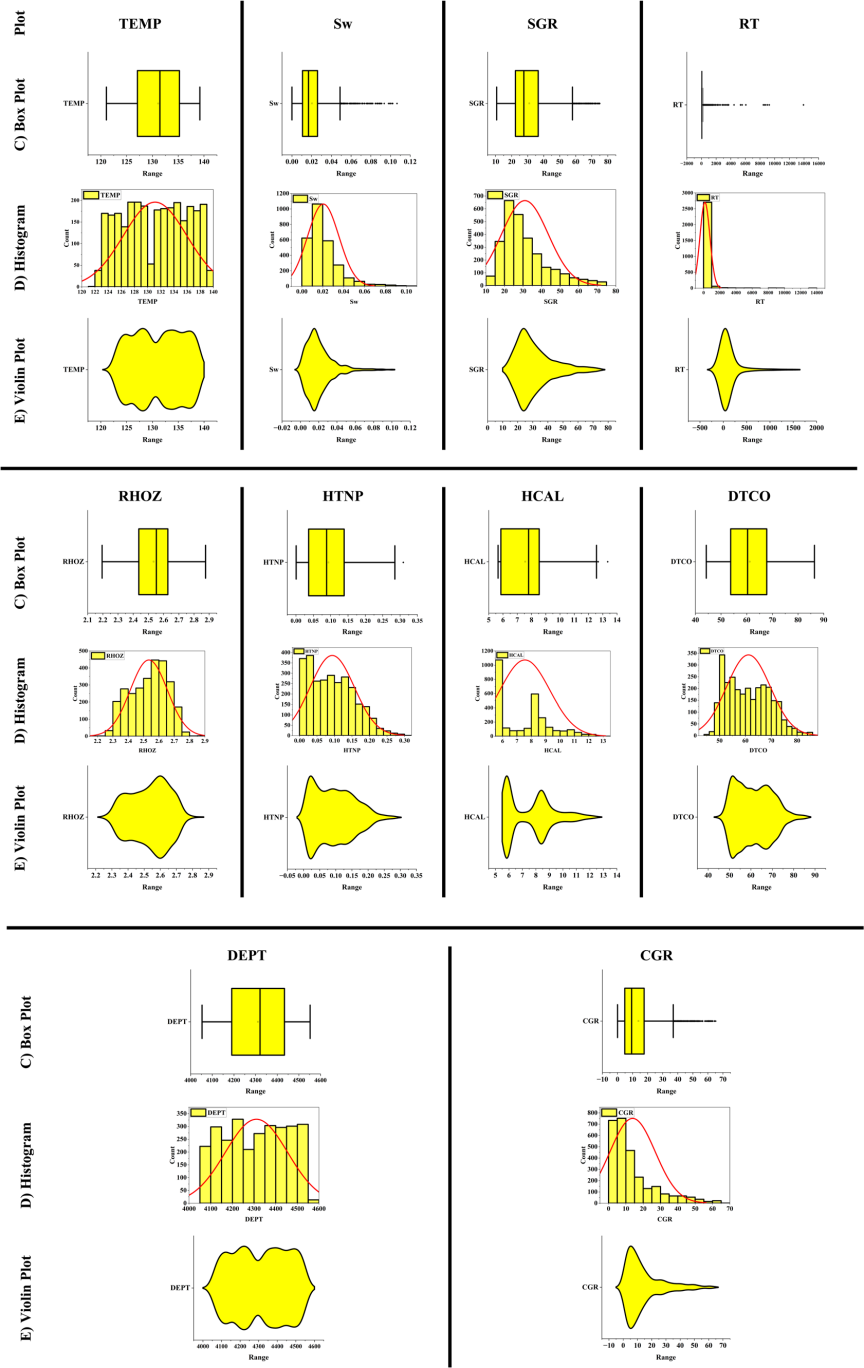
Specialized analysis of input data (After outlier removal). (**A**) Scatter plots & Histograms, (**B**) Heat map, (**C**) Box plots, (**D**) Histograms, (**E**) Violin plots.

In this paper, the outlier removal method was applied using a Gaussian-based approach to enhance data quality. To visualize the improvement in data quality, tools such as scatter plots, histograms, heatmaps, box plots, and violin plots were employed. The overall process of this study consists of three main stages:

- Initial data analysis without outlier removal (Fig. 1).
- Identification and visualization of outliers, where red triangles represent the outliers, and the symbol “*” denotes the remaining valid data points (Fig. 2).
- Analysis of data quality improvement after outlier removal (Fig. 3).

The Gaussian removal method, or outlier removal using the Gaussian distribution, is a statistical technique used to detect and eliminate outliers in a dataset. This method is based on the assumption that the data follows a normal distribution (Gaussian distribution), and data points that deviate significantly from this distribution are identified as outliers (Fig. 2).

The process of the Gaussian Removal method consists of three main steps. First, the mean $(\mu )$ and standard deviation $(\sigma )$ of the dataset are calculated to identify outliers. Then, data points are identified as outliers if their distance from the mean is greater than one or more times the standard deviation. This distance is usually defined as $\mu \pm k\alpha$, where $k$ is a constant value that determines the sensitivity to outliers. Finally, data points that fall outside this range are identified as outliers and removed.

Formulas for calculating mean and standard deviation:

1 $$\mu = \frac{1}{n}\mathop \sum \limits_{i = 1}^{n} x_{i}$$

2 $$\sigma = \sqrt {\frac{1}{n}\mathop \sum \limits_{i = 1}^{n} \left( {x_{i} - \mu } \right)^{2} }$$

where ${x}_{i}$ are the data points, $n$ is the number of data points, and $\mu$ is the mean of the data.

Gaussian removal equation:

Data points identified as outliers follow the following equation:

3 $$P\left( x \right) = \frac{1}{{\sigma \sqrt {2\pi } }}exp\left( { - \frac{{\left( {x - \mu } \right)^{2} }}{{2\sigma^{2} }}} \right)$$

where $P\left(x\right)$ is the probability density for data point $x$, $\mu$ is the mean of the data, $\sigma$ is the standard deviation of the data and $x$ are the data points.

Data points with very low probability values (i.e., significantly distant from the mean) are identified as outliers and removed.

A z-score threshold of ± 3.0 was selected for identifying outliers, corresponding to a 99.7% confidence interval under the assumption of a normal distribution. This threshold is commonly used in statistical analysis to balance the removal of anomalous values without discarding significant but valid data. Its selection was informed by prior studies and tailored to the size and variability of the dataset used in this study.

To scientifically demonstrate the improvement in data quality after outlier removal, various data analysis tools were used, including scatter plots, histograms, heatmaps, box plots, and violin plots. The results of these analyses are presented in Fig. 3.

### Machine learning methods

**Fig. 4:**
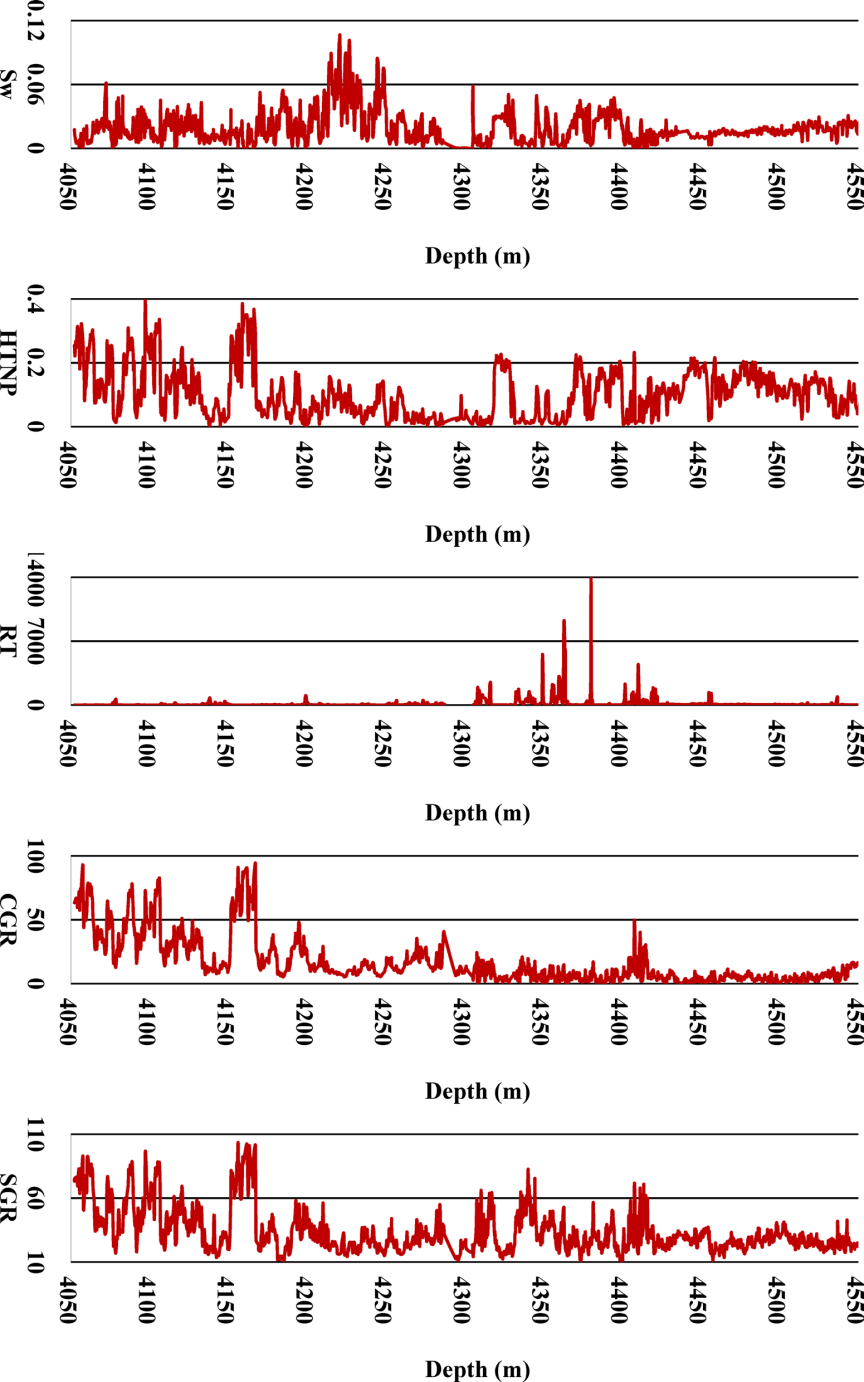

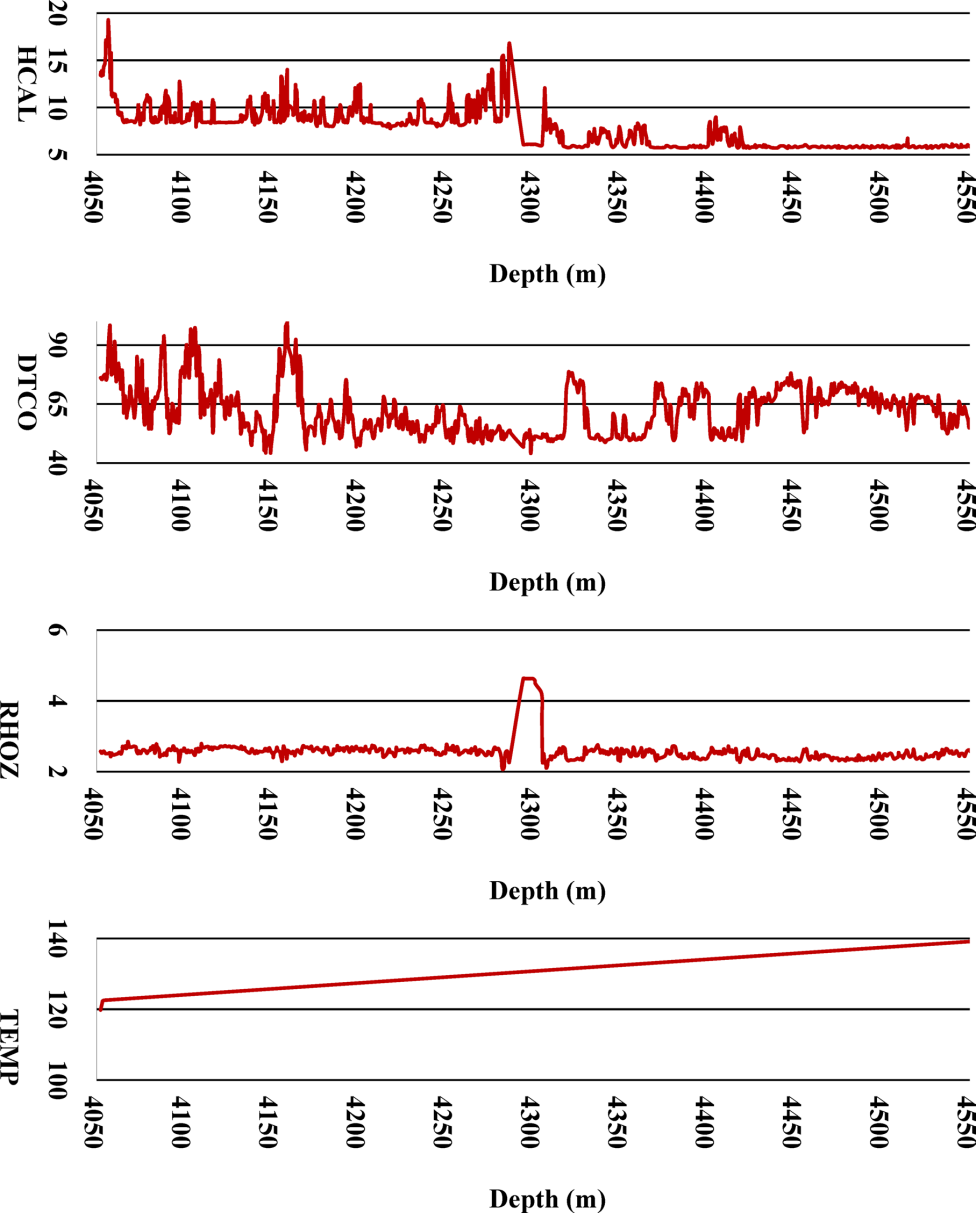
Desired Input Parameters for Developing ML Models.

To gain deeper insights into the vertical variation of petrophysical properties, Fig. 4 presents the depth-wise trends of all nine input parameters utilized for machine learning model development. By plotting these well-log features—such as porosity, resistivity, gamma ray, and sonic travel time—as functions of depth, this figure enables the identification of geological transitions, lithological changes, and anomalous zones. Understanding these depth-dependent behaviors is crucial for both feature engineering and improving model interpretability, particularly in heterogeneous formations. These trends also highlight zones of consistent property behavior, which can guide spatially-aware data partitioning and ensure robust training and testing procedures. Depth-wise distribution of the nine well-log input parameters used in the ML models (DEPT, HTNP, RT, CGR, SGR, HCAL, DTCO, RHOZ, and TEMP). These plots reveal the variation of petrophysical properties with depth, highlighting zones of heterogeneity, lithological changes, and potential data anomalies. This visualization aids in understanding reservoir behavior and in developing more geologically consistent and interpretable ML models.

The selection of the five machine learning algorithms—LR, SVM, RF, LSBoost, and Bayesian modeling—was based on their proven effectiveness in regression-based reservoir property prediction and their ability to capture both linear and nonlinear relationships with varying degrees of interpretability and computational efficiency. These models were chosen to cover a broad range of algorithmic families, including parametric (LR, Bayesian), ensemble (RF, LSBoost), and kernel-based approaches (SVM), allowing for a robust comparative analysis across different learning paradigms. While neural networks and deep learning methods have shown great potential in subsurface modeling, they typically require significantly larger datasets, extensive computational resources, and longer training times. Given the structure and volume of our dataset, as well as the objective of developing interpretable and computationally efficient models, we prioritized algorithms that balance performance with clarity and feasibility. Nonetheless, future work may explore deep learning models to further evaluate their predictive capacity in Sw estimation.

To mitigate the risk of data leakage arising from spatial or depth-wise correlation in well-log datasets, the data splitting strategy was revised to incorporate spatial separation. Data points were grouped based on well identifiers and depth intervals, ensuring that samples from the same well or adjacent depths were not simultaneously included in both training and testing sets. This spatially-aware partitioning strategy preserves the geological continuity of the data and provides a more reliable assessment of model generalization. Grouped cross-validation was employed to evaluate model performance under these constraints, thereby improving the robustness of the results.

#### Bayesian

**Fig. 5:**
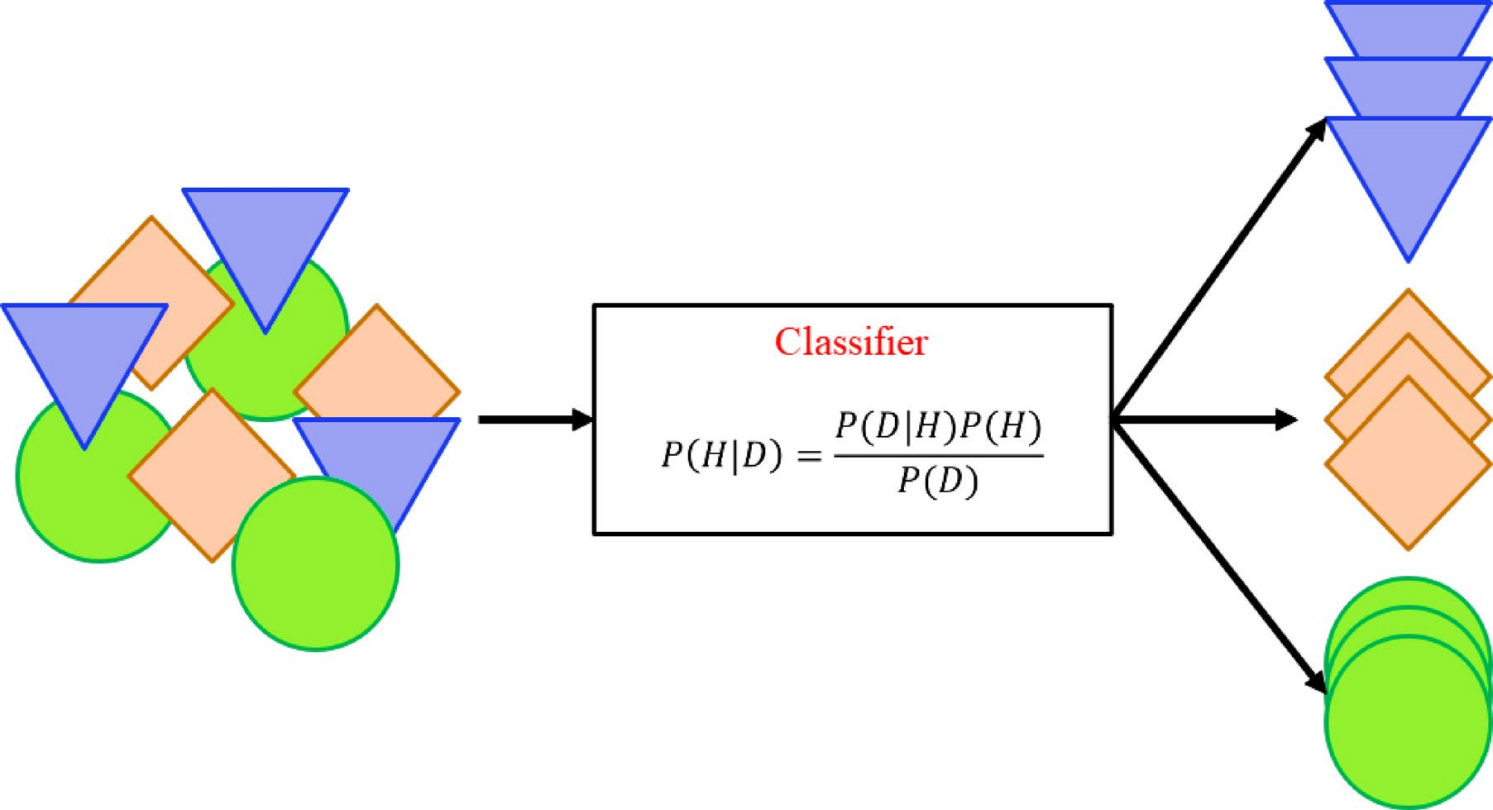
Bayesian algorithm.

The Bayesian approach is a fundamental technique in ML that leverages the principles of Bayesian probability to model and learn from data. This method integrates prior knowledge with evidence obtained from the data. At its core lies Bayes’ theorem, which defines the relationship between conditional probabilities (see Fig. 5). Bayes’ Theorem can be written as:

4 $$P\left( {H|D} \right) = \frac{{P\left( {D|H} \right)P\left( H \right)}}{P\left( D \right)}$$

In this equation, $P\left( {H|D} \right)$ represents the posterior probability, which indicates the likelihood of hypothesis $H$ given the evidence $D$. $P\left( {D|H} \right)$ is the likelihood, showing how probable the data $D$ is under the assumption that hypothesis $H$ is true. $P\left( H \right)$ is the prior probability, representing initial knowledge about hypothesis $H$, and $P\left( D \right)$ is the marginal likelihood, acting as a normalizing factor.

In machine learning, $H$ typically denotes the model or its parameters, while $D$ represents the training data. The goal is to estimate the posterior probability $P\left( {D|H} \right)$ to learn the model or its parameters. Bayesian methods are broadly categorized into parametric Bayesian learning and non-parametric Bayesian learning. In parametric learning, the model parameters are assumed to be fixed but unknown. For instance, if $\theta$ represents the model parameters, the posterior distribution is expressed as:

5 $$P\left( {\theta |D} \right) = \frac{{P\left( {D|\theta } \right)P\left( \theta \right)}}{P\left( D \right)}$$

Conversely, non-parametric Bayesian learning is utilized when the model structure or the number of parameters is not predefined. This method is often applied in models such as Gaussian processes or Bayesian clustering, where the complexity of the model dynamically adapts to fit the data.

One of the key applications of Bayesian methods in ML is prediction. Predictions are made using the posterior expectation. For example, the prediction of ${y}^{*}$ for a new data point ${x}^{*}$ is calculated as:

6 $$P\left( {y^{*} |x^{*} ,D} \right) = \smallint P\left( {y^{*} |x^{*} ,\theta } \right)P\left( {D|\theta } \right)d\theta$$

Here, $P\left({y}^{*}|{x}^{*},\theta \right)$ is the predictive probability of the output ${y}^{*}$ given the input ${x}^{*}$ and parameters $\theta$, and $P\left(D|\theta \right)$ represents the posterior distribution of the parameters. This integral is often computed using methods such as Monte Carlo sampling (MCMC) or other approximation techniques.

Bayesian methods are applied in various models, such as the Naive Bayes Classifier and Bayesian Networks. In the Naive Bayes Classifier, it is assumed that the features are independent of each other, and the probability of a class $C$ given features ${x}_{1}, {x}_{2}, \dots , {x}_{n}$ is calculated as:

7 $$P\left( {C|x_{1} , x_{2} , \ldots , x_{n} } \right) \propto P\left( C \right)\mathop \prod \limits_{i = 1}^{n} P\left( {x_{i} |C} \right)$$

On the other hand, Bayesian Networks leverage the causal relationships between variables. In these models, the nodes represent the variables, while the edges indicate the probabilistic dependencies connecting them.

In this study, the Bayesian method was implemented using a Gaussian prior distribution and the Markov Chain Monte Carlo (MCMC) sampling technique. A burn-in period of 100 iterations and 1000 posterior samples were used to ensure convergence. The model was configured for regression, with hyperparameters selected based on prior studies and refined through performance-based sensitivity analysis. The implementation aimed to evaluate the probabilistic estimation behavior of water saturation under uncertainty, while maintaining interpretability and robustness.

The Bayesian approach offers both strengths and limitations. Among its advantages are the ability to integrate prior knowledge with new data, the provision of probabilistic distributions instead of fixed values, and its suitability for situations with limited data. However, its main challenges include high computational complexity and sensitivity to the choice of the prior distribution.

#### Linear regression

Linear Regression is a fundamental and commonly applied supervised learning algorithm in machine learning. It aims to capture the relationship between independent variables (features) and a dependent variable (target) by fitting a straight-line equation to the observed data. The main goal is to estimate the dependent variable’s value based on the provided independent variables. The general mathematical formulation of LR is represented as:

8 $$y = \beta_{0} + \beta_{1} x_{1} + \beta_{2} x_{2} + \ldots + \beta_{p} x_{p} + \varepsilon$$

where, $y$ is the dependent variable, $x_{1} ,x_{2} , \ldots , x_{p}$ are the dependent variable, $\beta_{0}$ is the intercept, $\beta_{1} ,\beta_{2} , \ldots , \beta_{p}$ are the coefficients, and $\varepsilon$ is the error term. In matrix form, it can be written as:

9 $$y = X\beta + \varepsilon$$

Here, $y, X, \beta , and \epsilon$ are matrices and vectors representing the data, coefficients, and errors. The main goal of LR is to minimize the error between the predicted and observed values, which is typically measured using the Mean Squared Error (MSE). The formula for MSE is:

10 $$MSE = \frac{1}{n}\mathop \sum \limits_{i = 1}^{n} \left( {y_{i} - \hat{y}_{i} } \right)^{2}$$

where, ${y}_{i}$ represents the actual values, and ${\widehat{y}}_{i}$ represents the predicted values.

The parameters of the model $\left(\beta \right)$ are estimated using the Ordinary Least Squares (OLS) method. The OLS solution is derived as:

11 $$\beta = \left( {X^{T} X} \right)^{ - 1} X^{T} y$$

Once the model is trained, predictions for new data points are made using the following formula:

12 $$\hat{y} = x \cdot \beta$$

Here, $x$ is the feature vector of the new data point.

The Linear Regression model was employed using the OLS approach with L2 regularization (ridge regression). A regularization coefficient of α = 0.01 and convergence tolerance of 1e-4 were applied to prevent overfitting while preserving model simplicity. This model served as a baseline to evaluate the linear predictability of Sw from the selected input features and to compare against more complex nonlinear models.

#### Support vector machine

Support Vector Machines (SVMs) are a powerful supervised learning algorithm widely utilized for tasks such as classification, regression, and outlier detection. They perform well in high-dimensional spaces and can manage both linear and nonlinear classification problems. The key goal of an SVM is to determine a hyperplane that best separates data points belonging to different classes in the feature space.

The central idea of SVM is to determine a hyperplane that maximizes the margin between two classes. This margin is defined as the distance between the hyperplane and the nearest data points from each class, known as support vectors. For a training dataset represented as $\left\{\left({x}_{i}, {y}_{i}\right)\right\}$, where ${x}_{i}$ is the feature vector and ${y}_{i}\in \left\{-1, +1\right\}$ is the class label, the hyperplane is mathematically defined as:

13 $$w^{T} x + b = 0$$

where, $w$ represents the weight vector, $x$ is the input feature vector, and $b$ is the bias term. The hyperplane acts as the decision boundary, while the support vectors are the data points closest to this boundary.

For linearly separable data, SVM aims to find the optimal hyperplane that maximizes the margin between the two classes. The equations defining the margin boundaries are:

14 $$w^{T} x_{i} + b = + 1 for y_{i} = + 1$$

15 $$w^{T} x_{i} + b = - 1 for y_{i} = - 1$$

This method guarantees that the hyperplane maximizes the separation between the classes while keeping the nearest points (support vectors) at the margin’s boundary.

The SVM model was configured with a RBF kernel, selected for its ability to capture nonlinear relationships. The regularization parameter was set to 10 and the tolerance was fixed at 0.001, as determined through empirical testing and cross-validation. Feature scaling was performed using z-score standardization prior to training to ensure numerical stability and improve convergence. SVM showed superior performance, particularly in handling the nonlinear and high-dimensional nature of the dataset.

#### Least squares boosting

Least Squares Boosting (LSBoost) is a ML technique that combines boosting with least squares regression to enhance prediction accuracy. Primarily used for regression tasks, it can also be adapted for classification problems. LSBoost improves upon traditional boosting methods by focusing on minimizing the least squares error of the model. The process starts with a simple initial model, typically the mean of the target values. During each boosting iteration, residuals are calculated by finding the difference between the actual values and the model’s current predictions. A new weak learner, usually a decision tree, is then trained to predict these residuals, aiming to minimize the least squares error. The model is updated by incorporating the scaled predictions of this weak learner, with adjustments made via a learning rate. After a set number of iterations or once the model’s performance stabilizes, the final model is formed by combining the predictions from all weak learners.

The initial prediction is computed as:

16 $$\hat{y}_{0} = \frac{1}{N}\mathop \sum \limits_{i = 1}^{N} y_{i}$$

where, ${y}_{i}$ is the actual target value for the $ith$ instance and $N$ is the total number of instances.

At iteration $m$, the residuals are calculated as:

17 $$r_{i} \left( m \right) = y_{i} - \hat{y}_{i} \left( m \right)$$

where $\hat{y}_{i} \left( m \right)$ represents the prediction for the $ith$ instance at iteration $m$.

A weak learner is then trained on these residuals with the goal of minimizing the least squares error, which is represented as:

18 $$min\mathop \sum \limits_{i = 1}^{N} \left( {r_{i} \left( m \right) - f_{m} \left( {x_{i} } \right)} \right)^{2}$$

where $f_{m} \left( {x_{i} } \right)$ is the prediction of the weak learner for the $ith$ instance.

The model is updated by adding the predictions of the weak learner, scaled by a learning rate $\alpha$:

19 $$\hat{y}_{i} \left( {m + 1} \right) = \hat{y}_{i} \left( m \right) + \alpha f_{m} \left( {x_{i} } \right)$$

where $\alpha$ is the learning rate (also known as the shrinkage parameter).

After $M$ iterations, the final prediction is given by:

20 $$\hat{y}_{i} = \hat{y}_{0} + \mathop \sum \limits_{m = 1}^{M} \alpha f_{m} \left( {x_{i} } \right)$$

LSBoost was implemented using 50 boosting iterations with a learning rate of 0.1. Decision trees with a maximum depth of 3 were used as base learners to maintain model generalization and avoid overfitting. The model’s parameters were selected based on iterative testing and validation accuracy. LSBoost was specifically included to test the performance of additive ensemble learning in capturing complex dependencies within the Sw data.

#### Random forest

**Fig. 6:**
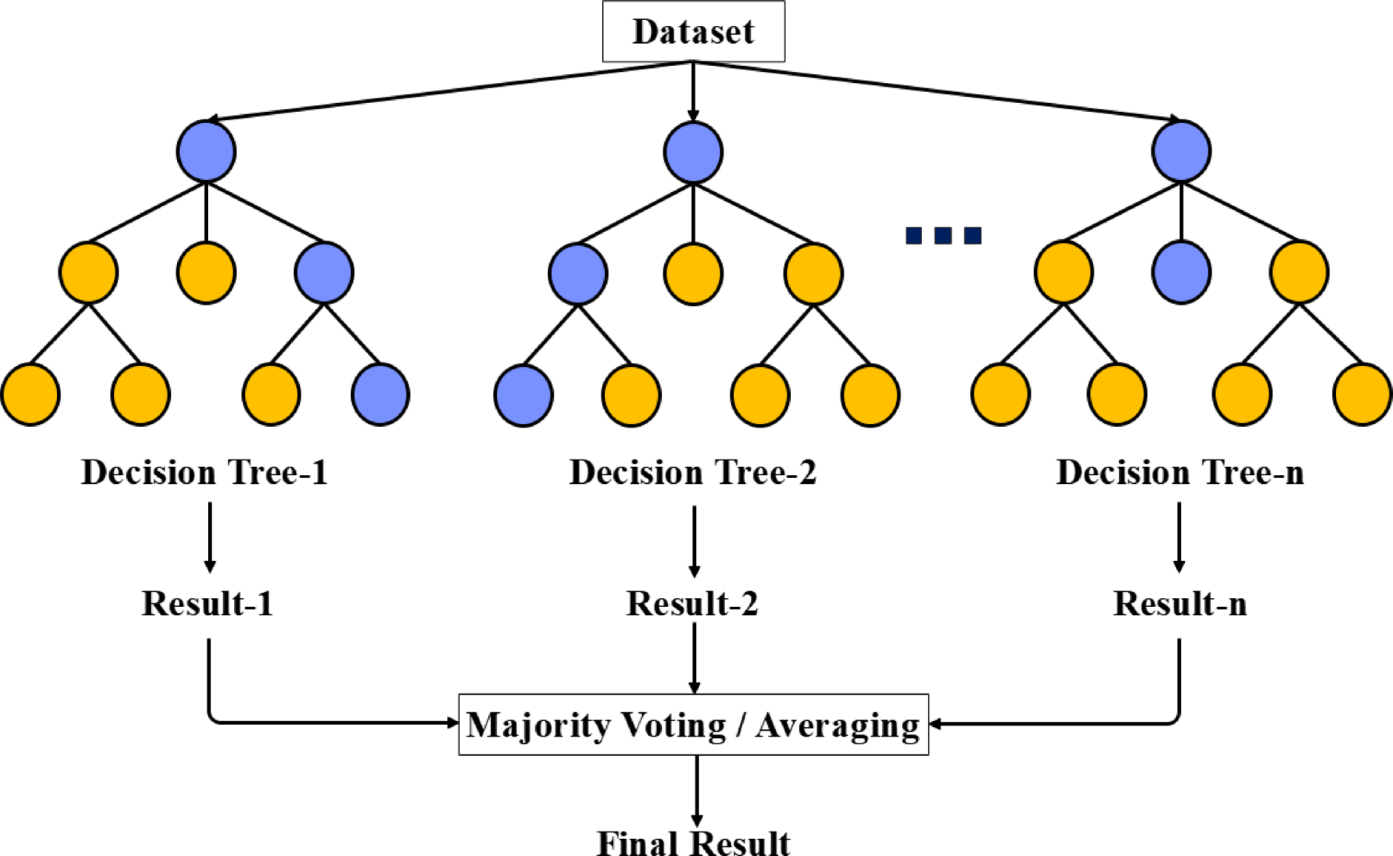
RF algorithm.

Random Forest (RF) is a popular ML method known for its effectiveness in classification and regression tasks, particularly when dealing with large and complex datasets. Its strength lies in its ability to reduce variance and avoid overfitting, making it a preferred option. RF is an ensemble technique that combines numerous decision trees, each trained independently on a random subset of the data. The overall prediction is derived by aggregating the outcomes from these individual trees (Fig. 6).

The RF algorithm begins by creating random samples from the training data using bootstrap sampling (sampling with replacement). Each decision tree is trained on a different random subset of the data. To ensure diversity among the trees, a random selection of features is made at each node of the trees. During prediction, the results from all trees are aggregated. For classification tasks, the final prediction is based on majority voting, while for regression tasks, the predictions are averaged.

In classification, the final prediction for a new sample $x$ is computed using the following formula, where $T_{1} , T_{2} , \ldots , T_{n}$ represent the decision trees and $C_{1} , C_{2} , \ldots , C_{n}$ are the possible classes:

21 $$Prediction \left( x \right) = \arg max\left( {\mathop \sum \limits_{j = 1}^{n} I\left( {T_{j} \left( x \right) = C_{i} } \right)} \right)$$

Here, $I$ is an indicator function that equals 1 if $T_{j} \left( x \right)$ equals $C_{i}$ and 0 otherwise.

For regression tasks, the final prediction for a new sample $x$ is calculated as the average of the predictions from all the decision trees:

22 $$Prediction \left( x \right) = \frac{1}{n}\mathop \sum \limits_{j = 1}^{n} T_{j} \left( x \right)$$

where $T_{j} \left( x \right)$ is the predicted value from decision tree $T_{j}$ for the sample $x$.

The RF model was constructed with 100 decision trees and a maximum tree depth of 10. Bootstrap sampling was enabled, and a minimum of two samples was required to split each node. This configuration was selected after several trials to balance performance and training efficiency. The model was used to evaluate the effectiveness of bagging-based ensemble techniques in reducing variance and improving prediction accuracy across heterogeneous input features.

Table 2 contains the key control parameters for various machine learning algorithms, which were carefully tuned and optimized to ensure the accuracy and reliability of the results. It includes the values and descriptions of relevant parameters for each algorithm, such as SVM, RF, LSBoost, Bayesian Method, and LR. These settings were selected based on preliminary testing, cross-validation, and best practices reported in the literature to optimize the performance of each algorithm.

**Table 2:** Control parameters for each algorithm.

Algorithm	Control parameter	Value/description
SVM	Kernel function	Radial basis function (RBF) kernel
	Regularization parameter	10
	Tolerance	0.001
RF	Number of trees	100 decision trees
	Maximum depth	10
	Minimum samples split	2
	Bootstrap sampling	Enabled (random sampling with replacement)
LSBoost	Number of iterations	50 boosting iterations
	Learning rate (shrinkage parameter)	0.1
	Base Learner	Decision trees with a maximum depth of 3
Bayesian	Prior Distribution	Gaussian prior
	Sampling algorithm	Markov chain Monte Carlo (MCMC)
	Burn-in period	100 iterations
	Number of samples	1000 samples
LR	Regularization	Ridge regression with L2 regularization (α = 0.01)
	Optimization algorithm	Ordinary least squares (OLS)
	Tolerance	1e-4 for convergence

## Results and discussion

### Data division into training and testing sets

At the beginning of this study, all the input parameters were thoroughly and comprehensively analyzed. Then, the input parameters, including SW, DEPT, HTNP, RT, CGR, SGR, HCAL, DTCO, RHOZ, and TEMP, were selected for calculations using ML algorithms in MATLAB software. After that, the input data was divided into two separate sets: one for training and the other for testing, to evaluate the performance of the algorithms. The proposed algorithms were executed for predicting Sw based on different training-to-test data ratios, and the accuracy of each method was calculated and presented graphically.

The random selection of input data based on these ratios can significantly affect the final accuracy of the algorithms. Therefore, each method was evaluated through ten independent runs, and the average R^2^ values obtained from these runs were reported as the final results. This evaluation approach provides an accurate representation of the performance and accuracy of each algorithm.

Table 3 reports the final R^2^ values for each layer in the range of 0.1 to 0.9. These values are independently calculated for each ML method and presented for the combined dataset of training/testing. Based on the results obtained from the training/testing rows, optimal models can be selected.

**Table 3:** Final R^2^ values for each layer across ML methods.

	Precision	R^2^-Bayesian	R^2^-LR	R^2^-SVM	R^2^-LSBoost	R^2^-RF
Test/train (All)	0.1	0.0981	0.0917	0.4804	0.4684	0.4524
	0.2	0.1996	0.1904	0.5799	0.5648	0.5463
	0.3	0.2927	0.2902	0.6822	0.6349	0.6637
	0.4	0.3962	0.3918	0.7860	0.7622	0.7656
	0.5	0.4936	0.4899	0.8842	0.8567	0.8657
	0.6	0.5909	0.5898	0.9913	0.9575	0.9684
	0.7	0.5887	0.5887	0.9924	0.9623	0.9731
	0.8	0.5890	0.5891	0.9938	0.9723	0.9767
	0.9	0.6825	0.6826	0.9957	0.9746	0.9874

The primary criterion for selecting the final results is the Test/Train ratio (All). In this context, the highest value from each method is chosen, and the corresponding regression plots are generated.

**Fig. 7:**
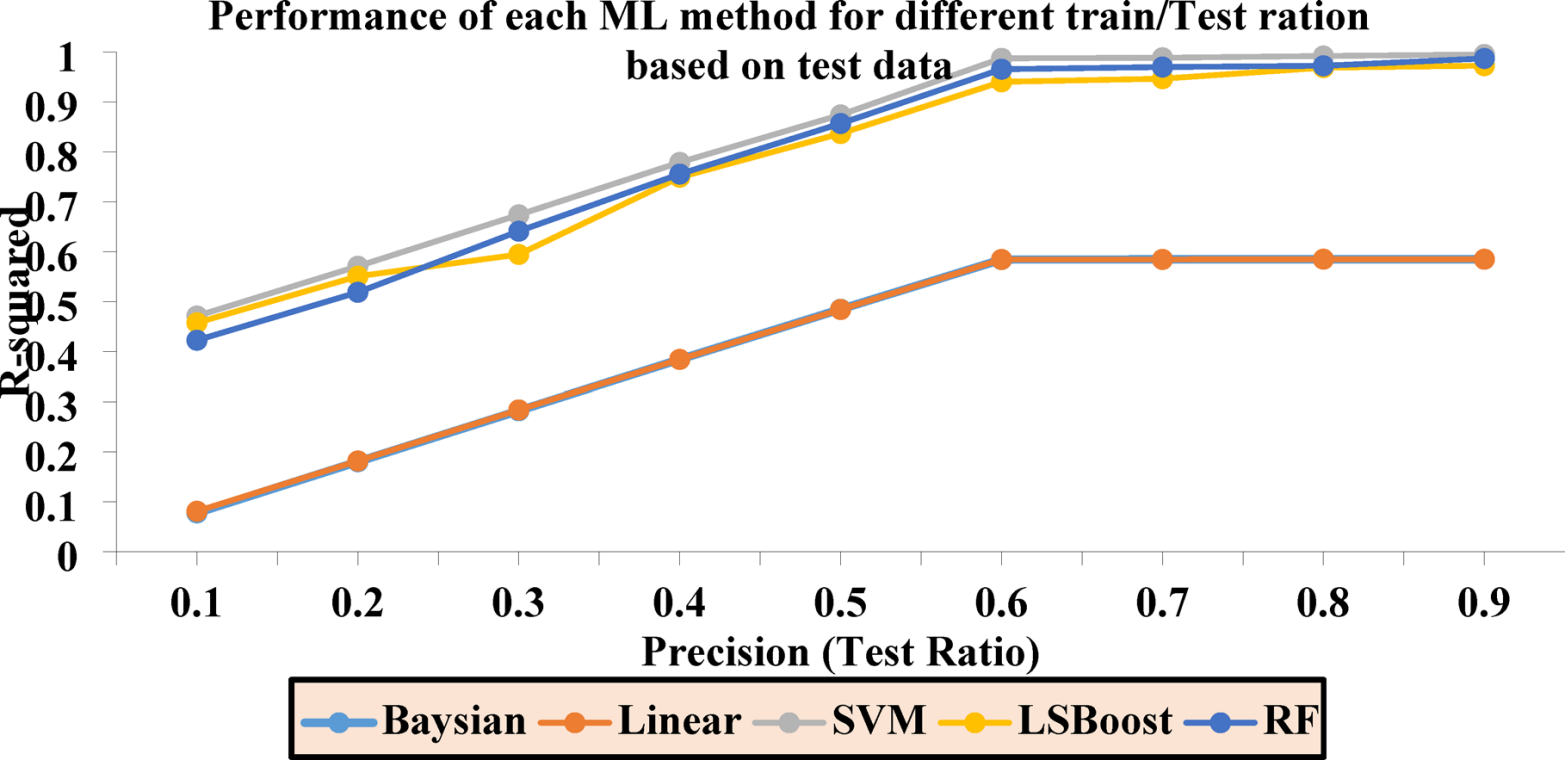
Accuracy of different ML methods in different percentages of training-to-test data based on test data.

**Fig. 8:**
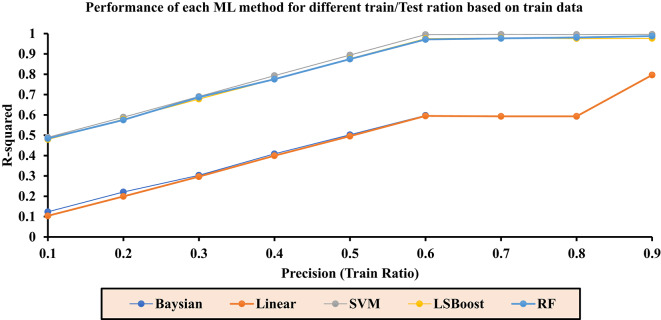
Accuracy of different ML methods in different percentages of training-to-test data based on train data.

**Fig. 9:**
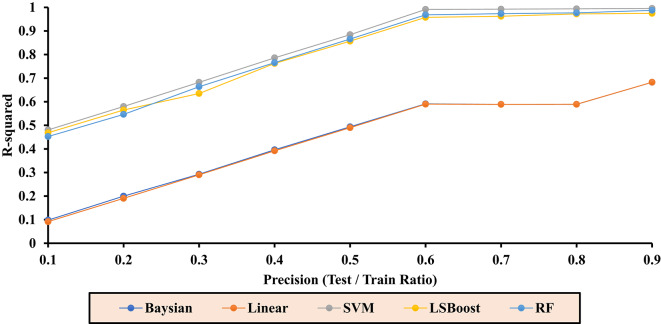
Accuracy of different ML methods in different percentages of training-to-test data.

To assess the optimal performance of Sw for each ML method, Fig. 7 illustrates the results based on the test data, while Fig. 8 displays the results using the training data. The accuracy of each method is evaluated, and the final selection of the best R^2^ value is referenced in the Test/Train Ratio section, as shown in Fig. 9.

Based on the results related to the water saturation coefficient (Sw) presented in Fig. 9, the highest accuracy is achieved when the ratio of training data to test data in the algorithms is set to Precision equal to 0.9.

To ensure the robustness of the proposed models and avoid issues such as overfitting or data leakage, a random subsampling cross-validation technique was employed. Specifically, the dataset was randomly divided into training and testing sets using multiple train-test splits (from 90/10 to 10/90), and each configuration was run ten independent times. The reported performance metrics, including R^2^, RMSE, and MAD, were averaged over these runs to minimize the impact of sampling variability. This repeated random splitting serves as an effective cross-validation method, especially for large datasets like ours (n = 30,660), ensuring the statistical stability and generalizability of the results.

While random splitting is commonly applied in machine learning tasks, it may introduce overly optimistic results when used with spatially correlated well-log data due to potential data leakage. To address this issue, a spatially separated data partitioning approach was implemented, ensuring that the training and testing datasets were mutually exclusive in terms of depth and well origin. This adjustment strengthens the reliability of the performance metrics and confirms the capability of the SVM model to generalize across different reservoir intervals.

### Performance of each method in the training and testing phases

The results from the regression analyses and R^2^ values are presented in graphical plots, which display data from both the training (Train) and testing (Test) sets together. These plots effectively illustrate the model’s fit to the data along with the final R^2^ value, providing a clear indication of the model’s performance. This approach is especially valuable for assessing the model’s accuracy in predicting test data and enables a direct comparison of its performance across both the training and testing datasets.

**Fig. 10:**
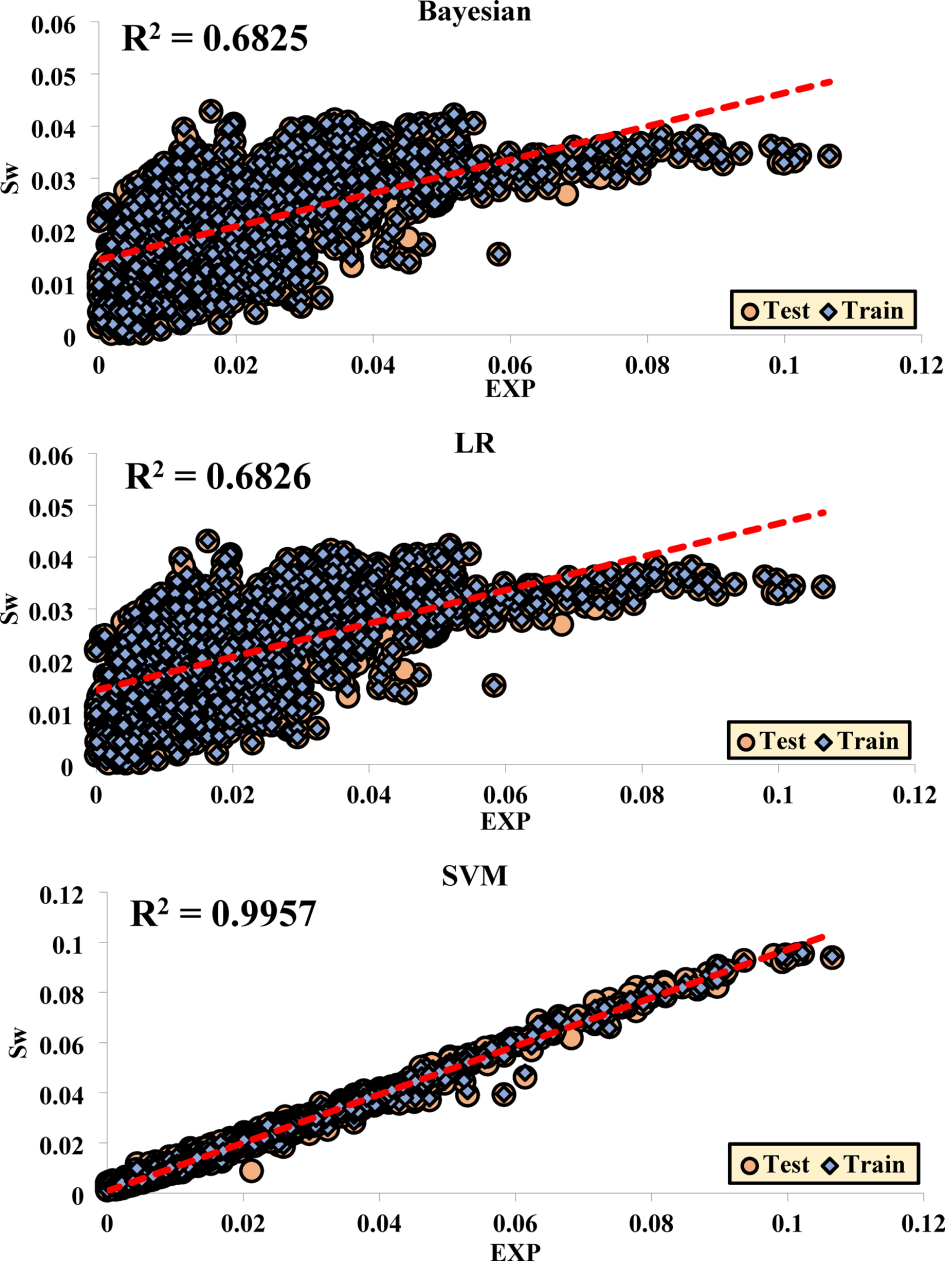

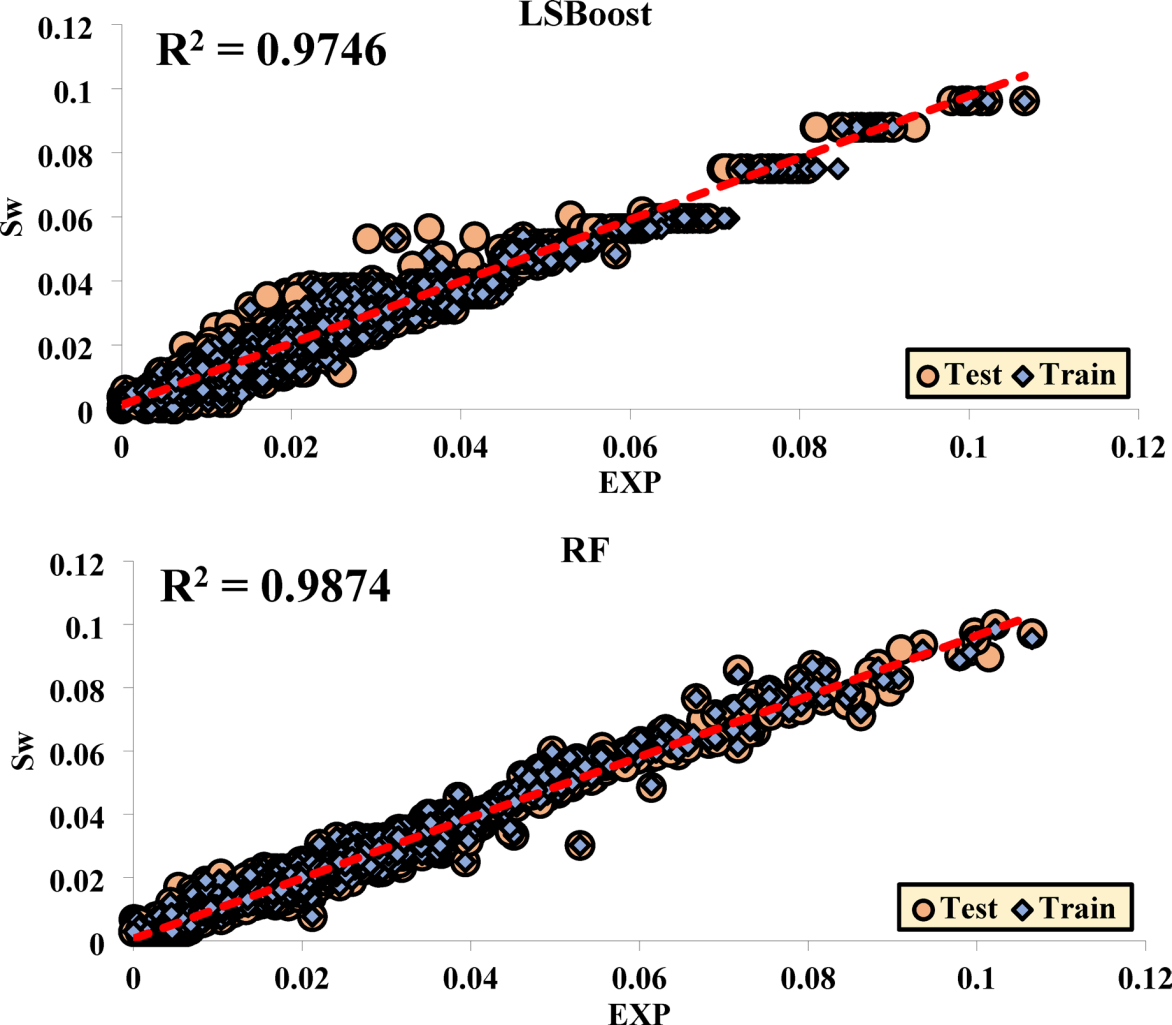
Regression of Different ML Methods for Sw.

Figure 10 shows the R^2^ values obtained from Sw.

Table 4 displays the regression equations developed to predict the output based on a given input variable. These equations were generated using various ML techniques to model and simulate the mathematical connections between input and output variables. In this study, the equations are tailored for analyzing and forecasting the behavior of different systems. By applying these methods, accurate models have been developed to define the relationship between inputs and outputs, which can be effectively utilized in decision-making and optimizing processes.

**Table 4:** The regression equations for each of the ML methods.

Equations		
Bayesian	Test	Output ~ = 0.35*Target + 0.013
	Train	Output ~ = 0.36*Target + 0.023
LR	Test	Output ~ = 0.35*Target + 0.013
	Train	Output ~ = 0.36*Target + 0.013
SVM	Test	Output ~ = 0.96*Target + 0.00077
	Train	Output ~ = 0.97*Target + 0.00077
LSBoost	Test	Output ~ = 0.98*Target + 0.00078
	Train	Output ~ = 0.96*Target + 0.00086
RF	Test	Output ~ = 0.96*Target + 0.00058
	Train	Output ~ = 0.97*Target + 0.00058

Although the regression slopes of LSBoost and RF models were close to those of the SVM model, a broader set of evaluation metrics was used to determine the optimal model. In particular, the SVM model yielded the highest R^2^ values and the lowest RMSE and MAD among all tested models. Furthermore, SVM showed consistent performance across ten random train-test splits, highlighting its stability and robustness. These factors collectively support the selection of SVM as the most effective model for Sw prediction in this study.

### Statistical criteria in measuring the accuracy of machine learning methods

In conclusion, the effectiveness and performance of the methods discussed in this article have been assessed and compared using statistical metrics such as the correlation coefficient and mean relative error. The following equations outline the calculation process for each of these statistical measures. Table 5 shows the performance results for each method.

**Table 5:** Plotted error values of ML methods.

	Method	R^2^ (Test)	R^2^ (Train)	R^2^ (All)	MAD (Test)	MAD (Train)	RMSE (Test)	RMSE (Train)
Sw	Bayesian	0.5850	0.7962	0.6825	0.009	0.009	0.012	0.012
	LR	0.5851	0.7963	0.6826	0.009	0.009	0.012	0.012
	SVM	0.9952	0.9962	0.9957	0.001	0.001	0.002	0.001
	LSBoost	0.9727	0.9764	0.9746	0.002	0.002	0.004	0.003
	RF	0.9872	0.9876	0.9874	0.002	0.002	0.002	0.002

Root Mean Square Error (RMSE) is a statistical measure used to assess the accuracy of predictive models. It quantifies the difference between the predicted values and the actual values. RMSE is commonly used to evaluate model performance by calculating the average squared error. The RMSE value can range from zero to infinity, with values closer to zero indicating higher model accuracy.

23 $$RMSE = \sqrt {\frac{1}{n}\mathop \sum \limits_{i = 1}^{n} \left( {y_{i} - \hat{y}_{i} } \right)^{2} }$$

The Mean Absolute Deviation (MAD) is a metric employed to evaluate the accuracy of predictive models by calculating the average of the absolute differences between observed and predicted values. A smaller MAD value indicates higher accuracy, while a MAD of zero indicates perfect alignment between the model’s predictions and the actual data. This metric is particularly useful for comparing the performance of various models and for fine-tuning them to achieve optimal outcomes.

24 $$MAD = \frac{1}{n}\mathop \sum \limits_{i = 1}^{n} \left| {y_{i} - \hat{y}_{i} } \right|$$

In this formula, ${y}_{i}$ represents the actual value for sample $i$, and ${\widehat{y}}_{i}$ is the predicted value for the same sample. $n$ denotes the total number of samples. This formula allows us to account for both positive and negative errors as absolute values, meaning all errors are calculated as positive values, which reduces the impact of large errors.

R^2^ is a statistical metric commonly used to assess the performance of prediction models and is regarded as a critical measure for evaluating model effectiveness. Also referred to as the coefficient of determination, R^2^ indicates the proportion of variance in the dependent variable that can be explained by the independent variables in the model’s predictions.

An R^2^ value near 1 implies that the model has effectively accounted for most of the variability in the dependent variable, with highly accurate predictions. Conversely, an R^2^ value near 0 suggests that the model has not explained much of the variance in the dependent variable, resulting in predicted values that significantly differ from the actual outcomes.

25 $$R^{2} = 1 - \frac{{\mathop \sum \nolimits_{i = 1}^{N} \left( {y_{i}^{Pred} - y_{i}^{exp} } \right)^{2} }}{{\mathop \sum \nolimits_{i = 1}^{N} \left( {y_{i}^{Pred} - average(y_{i}^{exp} } \right))^{2} }}$$

The exceptionally high R^2^ values observed for the SVM model—0.9952 for testing and 0.9962 for training—were consistently achieved across multiple random splits. Given the use of ten repeated random train-test divisions and performance averaging, these results are statistically robust and unlikely to be due to overfitting or data leakage. Furthermore, performance was validated across training, testing, and full datasets to ensure model generalizability.

**Fig. 11:**
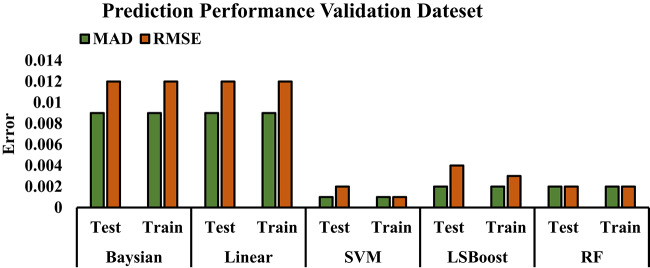
The error of each ML method.

Figure 11 visually presents the error data from Table 5, allowing for a straightforward comparison of the methods.

**Fig. 12:**
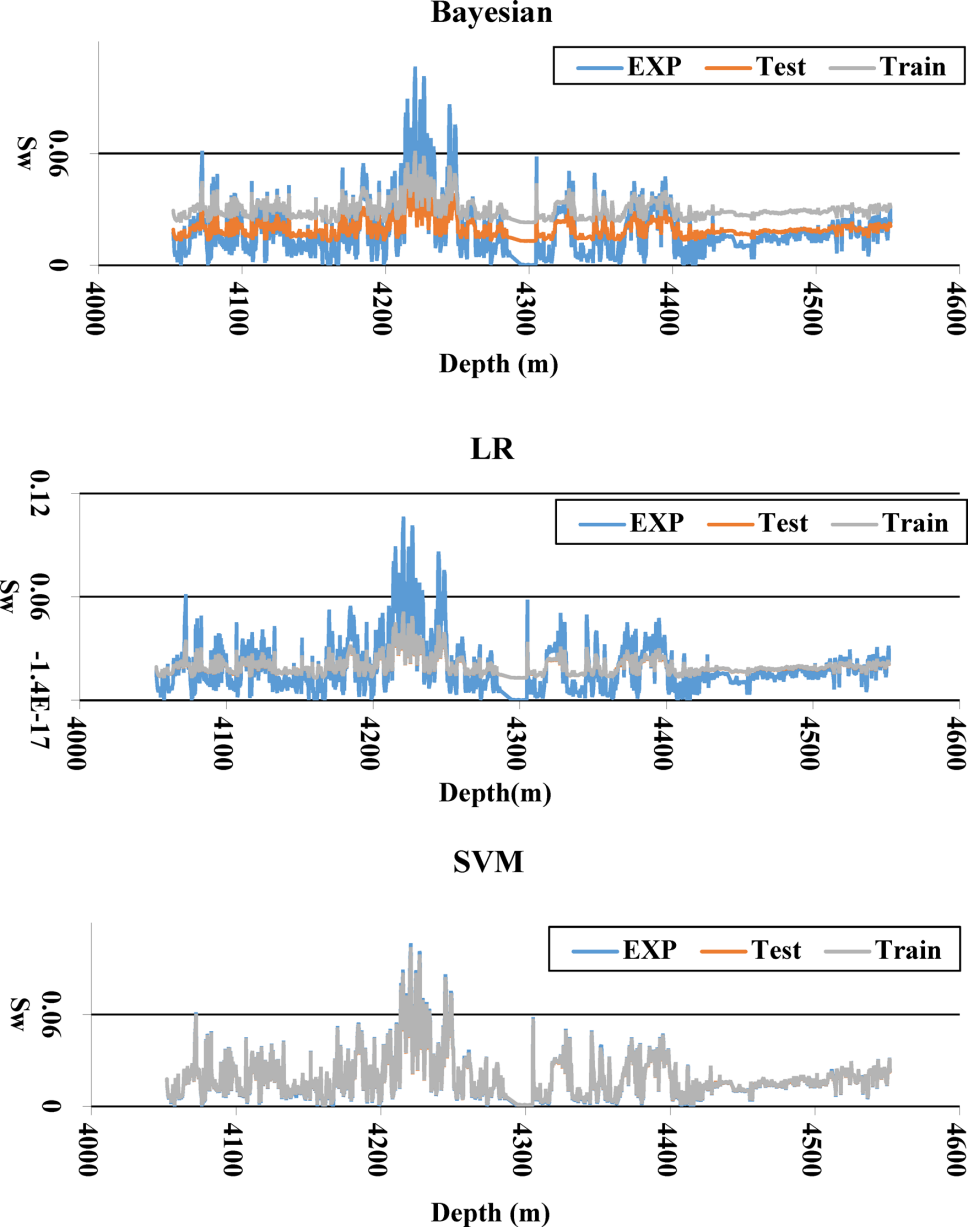

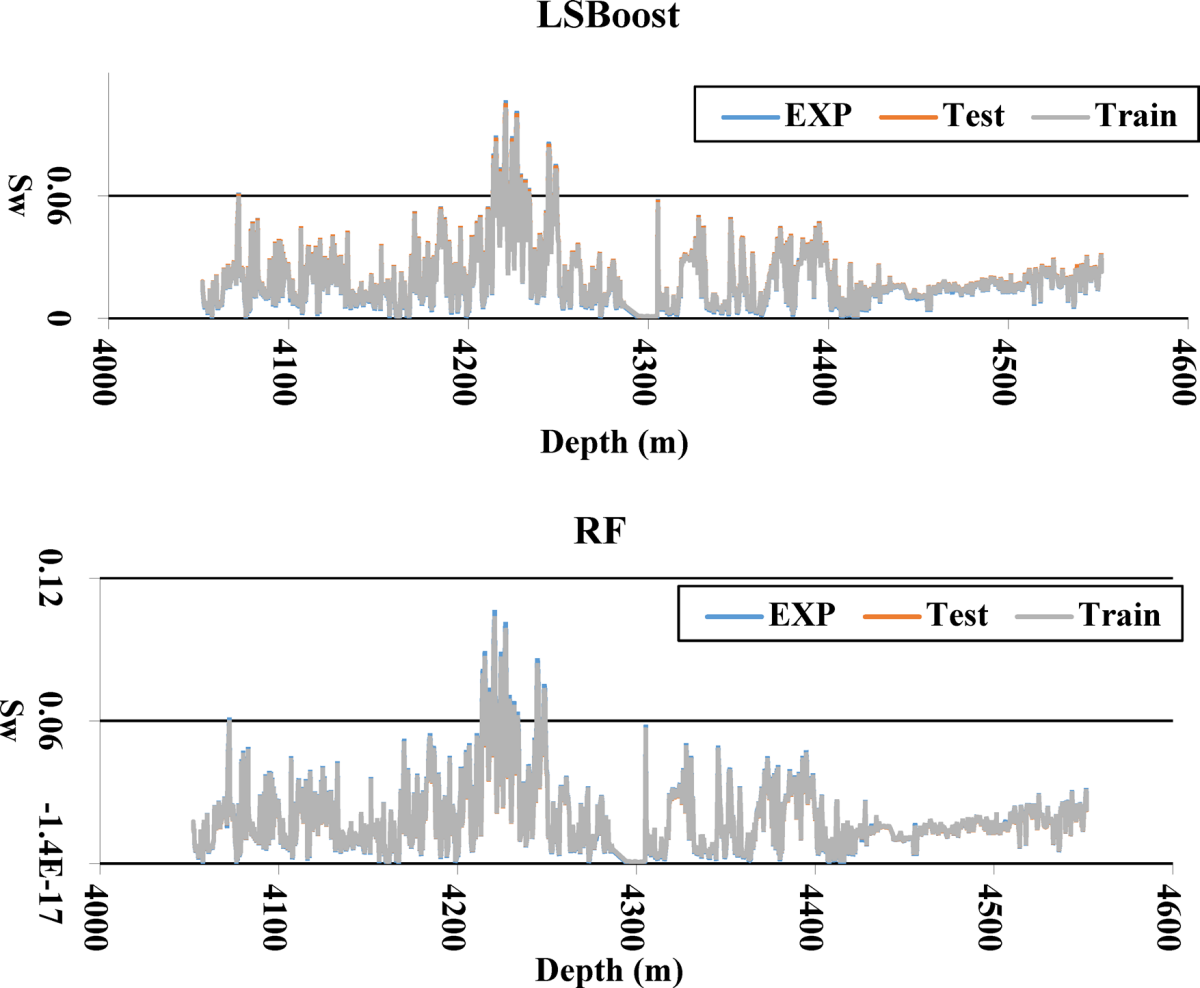
Comparison of training, testing, and experimental Sw data using different algorithms.

To thoroughly assess the performance of each algorithm in predicting water saturation, both the training and testing results were plotted together alongside the experimental data for a direct visual comparison. This approach enables a clear evaluation of the model’s accuracy and generalizability. As illustrated in Fig. 12, the SVM algorithm demonstrates superior performance compared to other models. The close alignment of the SVM’s training and testing curves with the experimental data indicates a high degree of predictive accuracy and minimal overfitting, suggesting that SVM is the most reliable and effective algorithm for estimating water saturation in this study.

Table 6 displays the results for the top-performing method (SVM) in predicting Sw characteristics. It includes several metrics used to assess the accuracy of the model, such as the following:

**Table 6:** Best results for each of the Sw.

Best method	R^2^ (test)	R^2^ (train)	R^2^ (All)	MAD (test)	MAD (train)	RMSE (test)	RMSE (train)
SVM	0.9952	0.9962	0.9957	0.001	0.001	0.002	0.001

This table highlights the performance of the SVM method as the most effective approach for data analysis. The following provides an explanation of the different criteria used to evaluate the model:

**Coefficient of determination (R** ^**2**^ **):**

- For test data (Test): A value of 0.9952 indicates very high prediction accuracy.
- For training data (Train): A value of 0.9962 shows that the model also performs very accurately during training.
- For all data (All): A value of 0.9957 reflects the overall balance and efficiency of the model.

**Mean absolute deviation (MAD):**

- For test data (Test): A value of 0.001 indicates a low average difference between predicted and actual values.
- For training data (Train): A value of 0.001 demonstrates higher accuracy during the training phase.

**Root mean squared error (RMSE):**

- For test data (Test): A value of 0.002 represents relatively low prediction error.
- For training data (Train): A value of 0.001 highlights the model’s very high accuracy on training data.

These results demonstrate that the RF method has been selected as the best approach, offering excellent performance in data prediction and analysis.

To account for the uncertainty and variability associated with machine learning predictions, especially critical in reservoir modeling, the performance of each algorithm was evaluated using ten independent runs across various training/testing data splits. For each run, the R^2^, RMSE, and MAD values were calculated and averaged to reduce the impact of random variations in data partitioning. Furthermore, the SD of these metrics was computed to quantify prediction variability. For instance, in the case of the SVM model under a 90/10 train-test split, the standard deviation of R^2^ across the ten runs was found to be ± 0.0012, and the RMSE varied within ± 0.0005. These low variability values confirm the statistical stability and reliability of the SVM model. The quantification of this uncertainty supports more robust decision-making and mitigates the risks associated with overfitting or overconfidence in model predictions (Table 7).

**Table 7:** Performance uncertainty for the SVM model over 10 runs.

Metric	Mean	Standard deviation (± SD)	Min	Max
R^2^ (test)	0.9952	± 0.0012	0.9936	0.9965
R^2^ (train)	0.9962	± 0.0009	0.9951	0.9974
RMSE (test)	0.0020	± 0.0005	0.0016	0.0025
RMSE (train)	0.0010	± 0.0003	0.0008	0.0013
MAD (test)	0.0010	± 0.0004	0.0007	0.0014
MAD (train)	0.0010	± 0.0003	0.0008	0.0012

While the results of this study demonstrate the high accuracy and reliability of ML techniques—particularly SVM—for estimating Sw, several limitations must be acknowledged. First, the model’s generalizability to other geological formations has not been fully tested. The dataset used was sourced from sandstone-dominated reservoirs in southwestern Iran, and its application to different lithologies (e.g., carbonates, fractured systems) may require retraining or adaptation of the model due to different petrophysical behaviors and data characteristics.

Second, although the Gaussian outlier removal method improved data quality, it relies on the assumption of normal distribution of input variables, which may not always hold true in geologically complex reservoirs. Over-removal of edge-case but valid data points may lead to loss of important geophysical signals or rare lithological patterns.

Third, the study employed log-derived data with certain limitations in resolution and granularity. Well logs are inherently limited in vertical sampling density and may not capture thin-bed effects or small-scale heterogeneity, which could influence prediction accuracy. The impact of measurement noise and tool calibration errors also needs further consideration.

Moreover, this study used static ML models without incorporating uncertainty propagation or dynamic reservoir behavior. While standard deviation across runs was quantified, more advanced uncertainty quantification techniques—such as Bayesian inference with probabilistic outputs or Monte Carlo-based scenario testing—could offer deeper insights.

## Conclusions

The accurate estimation of water saturation (Sw) is a critical component in optimizing oil recovery strategies and improving reservoir characterization. This study demonstrated the effectiveness of machine learning (ML) techniques in predicting Sw by leveraging multiple well-log parameters. Among the five ML models evaluated—Linear Regression (LR), Support Vector Machine (SVM), Random Forest (RF), LSBoost, and Bayesian methods—the SVM model exhibited the highest accuracy. With an R-squared value of 0.9952 for test data and 0.9962 for training data, along with exceptionally low RMSE values (0.002 for test data and 0.001 for training data), SVM outperformed other models in handling complex petrophysical relationships.

The integration of the Gaussian outlier removal technique significantly improved data quality, leading to more reliable model predictions. Performance metrics such as R-squared, Mean Absolute Deviation (MAD), and RMSE confirmed the robustness of the SVM model, demonstrating its ability to generalize well across both training and test datasets. The consistency of R-squared across training (0.9962), test (0.9952), and overall data (0.9957) suggests a well-balanced and efficient model. Additionally, the exceptionally low MAD (0.001) and RMSE values reinforce the reliability of this approach.

These findings highlight the potential of ML, particularly SVM, as a robust alternative to conventional Sw estimation methods, which are often constrained by core-derived parameters and idealized assumptions. The application of ML-driven Sw prediction can enhance reservoir evaluation, optimize production strategies, and contribute to more efficient hydrocarbon recovery. Future studies could further refine this approach by integrating additional well-log data, considering advanced deep learning techniques, and applying these models to a broader range of reservoir conditions to improve generalizability.

## Data Availability

The datasets used and/or analyzed during the current study available from the corresponding author on reasonable request.

## Abbreviations

EOR: Enhanced oil recovery
BMA: Model averaging
GLM: Generalized linear models
LR: Linear regression
LSBosost: Least squares boosting
MAD: Mean absolute deviation
ML: Machine learning
MSE: Mean squared error
MCMC: Markov Chain Monte Carlo
RF: Random forest
RMSE: Root mean square error
SVM: Support vector machine
SGAM: Smooth generalized additive models
Sw: Water saturation
TCP: Total correct percentages
